# Prisoners of the Body: The Link Between Interoception and Sleep Paralysis

**DOI:** 10.1111/jsr.70393

**Published:** 2026-06-12

**Authors:** Giuseppe Forte, Danilo Giaccari, Francesca Favieri, Maria Casagrande

**Affiliations:** ^1^ Department of Dynamic Clinical Psychology and Health “Sapienza” University of Rome Rome Italy; ^2^ Department of Psychology University G. d'Annunzio of Chieti‐Pescara Chieti Italy

**Keywords:** interoceptive sensitivity, pre‐sleep arousal, REM parasomnia, sleep paralysis, somatic alertness

## Abstract

Sleep paralysis (SP) is a REM sleep parasomnia characterized by transient motor paralysis during sleep onset or awakening. It is a terrifying experience often accompanied by vivid hallucinations. Although it is classically linked to narcolepsy, isolated SP in healthy individuals offers a valuable model to investigate the interaction between REM sleep physiology and conscious awareness. In a sample of 210 adults from the Italian population, we examined the prevalence, phenomenology and cognitive–emotional landscape of SP. Importantly, this study provides preliminary evidence to integrate measures of interoception to investigate how body awareness might influence this experience. One out of four participants reported at least one episode of SP. The most common sensations echoed REM physiology, including the inability to move or speak, tingling and sealed eyelids. However, despite this paralysis, the mind conjured up vivid cognitive scenes and the felt presence of another being, as well as an overpowering fear of death. Contrary to earlier findings, SP was not tied to poorer sleep quality or chronic anxiety. Instead, it was associated with insomnia symptoms and hyper‐attuned body awareness. Individuals experiencing SP showed greater interoceptive sensitivity, noticing subtle bodily cues and regulating attention and emotion through them. This convergence between sleep physiology and interoceptive awareness suggests that SP may emerge where the boundaries among body, sleep and consciousness become blurred, a dissociated state in which REM‐related atonia coexists with preserved or re‐emerging conscious awareness. These findings suggest potential avenues of interoception as a target for future investigations into parasomnias.

## 1 Introduction

Sleep paralysis (SP) is a transient, generalized inability to move or speak during sleep onset or upon awakening (Nielsen and Zadra 2011; Sateia 2014). It can manifest as Isolated Sleep Paralysis (ISP), or as a part of the clinical narcolepsy tetrad, where it has been reported in up to 50% of patients (Frauscher et al. 2013). Typically occurring during rapid eye movement (REM) sleep, SP is classified as a REM parasomnia (Sateia 2014) in which the physiological muscle atonia of REM sleep prevents movement in response to dreams. Consequently, the dissociation between perceived wakefulness and motor paralysis arises when the individual becomes consciously aware during this state of atonia.

SP is characterized by feelings of unpleasantness and fear due to the experience of a complete inability to move despite subjective alertness, especially when it occurs for the first time. This distress is often compounded by hypnagogic or hypnopompic hallucinations, with themes that differ across cultures but commonly involve sinister intruders, sensations of suffocation or chest pressure and physical and sexual assaults (Ho¨Gl and Iranzo 2017; Fukuda 2005). Hallucinations may contribute to the misdiagnosis of SP as a psychotic disorder. Although specific electrophysiological patterns have been described in SP, current evidence suggests a more complex, heterogeneous profile rather than a universally consistent signature. In particular, studies conducted during SP episodes (e.g., Mainieri et al. 2021) have reported mixed alpha and theta activity, whereas investigations conducted outside actual episodes (e.g., Kliková et al. 2021) have identified increased beta activity. These differences likely reflect methodological variability and distinct physiological states captured across studies.

The prevalence of SP in the overall population is estimated at around 7.6%, with a higher rate in university students, which can reach 37.5% (Farooq and Anjum 2023; Colombo and Cellini 2024), or in psychiatric patients, with an incidence of 30% (Johnson 2023). However, most epidemiologic data were derived from small, specific samples and the prevalence of this disorder in the general population remains unclear due to the terminology used to describe the phenomenon or the reports provided (Hefnawy et al. 2024; Fukuda 1993). Similarly, aside from its well‐documented association with narcolepsy, the relationship between SP and other disorders has received limited attention.

However, some evidence suggests that some risk factors can contribute to the onset of SP, such as anxiety disorders (Otto et al. 2005; Paradis et al. 2009; Jalal and Hinton 2015; Denis and Poerio 2016; Bell et al. 1984; Denis et al. 2018), stress and exposure to traumatic events (Jalal and Hinton 2015; McNally and Clancy 2005; Mellman et al. 2007), poor sleep quality (Denis et al. 2018; Sharpless and Barber 2011) and sleep disturbances (Hefnawy et al. 2024), as insomnia (Szklo‐coxe et al. 2007; Ohayon et al. 1999). Furthermore, SP appears to be associated with pre‐sleep arousal, and increased arousal precedes SP episodes (Takeuchi et al. 2002). This aspect suggests a role of physiological and bodily components of sleep, which are still poorly understood. What role does the body play, and how does the ability to interpret body sensations contribute to the experience of SP? Interoception, the process through which the nervous system perceives, interprets and integrates internal bodily signals (Khalsa et al. 2018), may play a fundamental role in shaping SP episodes. Altered interoceptive processing could contribute to the characteristic sensations of chest pressure, suffocation and the presence of an external entity commonly reported during SP episodes. Accordingly, investigating the role of interoception in SP could provide new insights into how the brain integrates bodily information during altered states of consciousness, potentially paving the way for targeted therapeutic interventions.

Building on these premises, this study aims to estimate the prevalence of SP and to identify its psychological and interoceptive correlates. Specifically, we examined how individual differences in sleep‐related and psychological variables contribute to the onset and phenomenology of SP. Particular attention was devoted to aspects of sleep quality—especially symptoms of insomnia—to clarify how disturbances in sleep continuity may increase vulnerability to SP episodes. Despite the growing body of research on SP, the role of interoceptive sensitivity has never been systematically examined. Likewise, no studies to date have addressed the relationship between SP and emotional regulation processes. The central innovation of the present work lies in integrating these two dimensions, offering a novel framework for understanding how body awareness and affective regulation may interact in shaping the experience and onset of SP. We therefore expect that individuals reporting SP will display greater interoceptive sensitivity, particularly in domains of body awareness and attentional regulation, as well as less adaptive emotional regulation strategies. In sum, this study aimed to examine the interplay among REM‐related physiology, conscious awareness and emotional processes in SP.

## 2 Materials and Methods

### 2.1 Participants

A total of 213 Italian adults voluntarily participated in the study. They were recruited through online advertisements and social media (WhatsApp, Telegram, Facebook, Instagram, Reddit). Inclusion criteria required participants to be 18 years of age or older and fluent in Italian. Exclusion criteria referred specifically to severe neurological or psychiatric conditions that could impair comprehension or significantly alter sleep perception. Self‐reported lifetime conditions were not used as exclusion criteria and were retained for descriptive purposes. After data screening, three participants were excluded due to ambiguous descriptions of their SP experience, resulting in a final sample of 210 individuals (64.3% female; mean age = 43.9 ± 14.5 years). A post hoc power analysis (G*Power 3.1) indicated that the sample size provided sufficient power (1 − β > 0.80) to detect medium effect sizes (Cohen's *d* = 0.5) at *α* = 0.05.

### 2.2 Measures

The *Unusual Sleep Experience Questionnaire* (USEQ; Paradis et al. 2009; Italian version: Colombo and Cellini 2024) was adopted and adapted to describe SP through self‐reported experiences, both quantitatively and qualitatively. The presence or absence of SP was determined by presenting each participant with a description of SP: ‘Some people have had the experience upon going to sleep or awakening when they were unable to move their arms or legs or speak, even though they wanted to do so. Have you ever had this experience?’ Participants who reported having experienced a similar event in their lives completed the questionnaire; otherwise, they moved on to the subsequent questionnaires. As a double control check, each participant had to describe their most vivid SP experience: ‘Please think about your most vivid experience and describe the experience in your own words (if you had only one episode of this experience, just describe that one)’. The questionnaire collected information about the frequency, duration, age of onset and events surrounding the SP episode. Examples of items include: ‘During an episode [of SP], how long are/were you usually unable to move or speak?’ and ‘Do/did you usually have the experience [of SP] when falling asleep or upon awakening?’ Some items quantified the intensity of subjective discomfort, physical symptoms and cognitive perceptions associated with the SP episodes. These items include: ‘People feel as though they are being smothered. Has this happened to you?’ and ‘People experience a feeling of presence in the room (‘Feeling of presence’ is the awareness of something with you in the room, even though you may not be able to see or hear anything). Has this happened to you?’

The USEQ was adapted to include additional items aimed at capturing specific aspects of SP phenomenology that were directly relevant to the study objectives and have been highlighted in previous literature, particularly fear intensity and sleep position during episodes (‘If you fall asleep again in the same position in which you had the experience, does it happen again?’, ‘In the first episodes, the level of fear was’ and ‘In the most recent episodes, the level of fear was’).

*Pittsburgh Sleep Quality Index* (PSQI; Buysse et al. 1989; Italian version: Curcio et al. 2012): a 19‐item questionnaire to measure sleep quality over the previous month. The questionnaire assesses seven clinically derived sleep difficulty domains (sleep quality, sleep latency, sleep duration, habitual sleep efficiency, sleep disturbance, use of sleep medications and daytime dysfunction). Taken together, these sleep domains converged into a single factor of global sleep quality. A global PSQI score greater than 5 is typically considered indicative of significant sleep disturbance, corresponding to poor quality in at least two components or moderate difficulty in more than three (Buysse et al. 1989). The Italian version of the PSQI has demonstrated good internal consistency, with Cronbach's *α* = 0.83 (Curcio et al. 2012).

*Disturbing Nighttime Behaviours* (PSQI‐A; Germain et al. 2004; Italian version: Palagini et al. 2016): this seven‐item subscale assesses PTSD‐related disturbing nighttime behaviours during the past month (e.g., nightmares, night terrors, or sudden awakenings with fear). Items are rated on a 4‐point Likert scale (0 = ‘not during the past month’ to 3 = ‘three or more times a week’). Higher scores indicate greater frequency of disturbing behaviours. The Italian version showed good internal reliability (Cronbach's *α* = 0.73).

*Insomnia Severity Index* (ISI; Italian version: Castronovo et al. 2016): a self‐assessment tool comprising seven items that evaluates the patient's perception of insomnia over the previous 2 weeks, rated on a five‐point Likert scale. It measures subjective symptoms and daytime consequences of insomnia, as well as the degree of discomfort associated with these difficulties. A higher score indicates a more severe form of insomnia. ISI also allows for the assessment of severity, impact and satisfaction. The questionnaire total score is divided as follows: 0–7, no significant insomnia; 8–14, subthreshold insomnia; 15–21, moderate insomnia; and 22–28, severe insomnia (Morin et al. 2011). The Italian version exhibits good internal reliability (Cronbach's *α* = 0.75).

The *Pre‐Sleep Arousal Scale* (PSAS; Nicassio et al. 1985; Italian version: Palagini et al. 2016) is a 16‐item self‐report questionnaire used to assess pre‐sleep arousals in their somatic (eight items) and cognitive (eight items) aspects experienced at bedtime while trying to fall asleep. Each item is rated on a five‐point Likert scale (1 = not at all; 5 = extremely). Higher scores indicate greater arousal. We adopted pathological cut‐off scores of 14 and 20 for the somatic and cognitive subscales, respectively (Puzino et al. 2020). Cronbach's alpha was 0.87 for the cognitive scale and 0.83 for the somatic scale.

The *State–Trait Anxiety Inventory* (STAI; Spielberger 1983; Italian version: Pedrabissi and Santinello 1989) is a self‐reported questionnaire designed to measure anxiety in adults. The instrument consists of two subscales, the first assessing anxiety as a transitory situational state and the second as a relatively constant personality trait (Gaudry et al. 1975). We used the STAI‐T (X‐2) subscale of the Italian adaptation of this questionnaire that assesses trait anxiety. The subscale consists of 20 items. Participants rate each item on a 5‐point Likert scale ranging from 1 (*not at all*) to 5 (*extremely*) to indicate the extent to which the behaviour described in the question is typical for them. This measure has demonstrated excellent psychometric properties (Gaudry et al. 1975). The Cronbach's alpha coefficient in this study was 0.91.

The *Perceived Stress Scale* (PSS; Cohen et al. 1983; Italian version: Mondo et al. 2019) is one of the most widely used self‐report questionnaires for measuring perceived stress. Based on Lazarus's theory of stress appraisal (Lazarus 1966; Lazarus and Folkman 1984), this instrument measures the degree to which aspects of one's life are perceived as uncontrollable, unpredictable and overloaded (Wang et al. 2011). The PSS assesses subjects' thoughts and feelings about stressful events that occurred in the month preceding the survey. The original version of the PSS consisted of 14 items (PSS‐14), including seven negatively stated and seven positively stated items. Two shorter forms of the PSS are available: a 10‐item form (PSS‐10), consisting of six negatively stated items and four positively stated items and a 4‐item form (PSS‐4), comprising two negatively stated items and two positively stated items.

For our study, we used the Italian version of PSS‐10 developed by Fossati (2010), which was tested by Mondo et al. (2019) and yielded an overall Cronbach's alpha of 0.74 (0.84 and 0.72 for the negative and positive subscales, respectively).

The *Multidimensional Assessment of Interoceptive Awareness* (MAIA; Mehling et al. 2012; Italian version: Calì et al. 2015) is a self‐administered questionnaire developed to measure eight dimensions of interoceptive sensitivity. It is composed of 32 items on a six‐point Likert scale (from 0—*never* to 5—*always*), the sum of which provides the total score. The eight dimensions derive from groups of specific items that refer to (1) noticing, the awareness of unpleasant, pleasant and neutral bodily sensations; (2) not‐distracting, the tendency to ignore or distract oneself from sensations of pain or discomfort; (3) not‐worrying, emotional distress or worry with sensations of pain or discomfort; (4) attention regulation, the ability to sustain and control attention to bodily sensations; (5) emotional awareness, the awareness of the connection between bodily sensations and emotional states; (6) self‐regulation, the ability to regulate psychological distress through attention to bodily sensations; (7) body listening, actively listening to the body to gain insights; (8) trusting, perceiving one's body as safe and reliable. The validated Italian version of Calì et al. (2015) has been shown to have Cronbach's alpha values ranging from 0.53 to 0.80.

The *Emotional Regulation Questionnaire* (ERQ; Gross and John 2003; Italian version: Balzarotti et al. 2010) is a 10‐item self‐report questionnaire consisting of two scales that correspond to two distinct emotion regulation strategies: cognitive reappraisal (6 items) and expressive suppression (4 items). The 10 items are rated on a 7‐point Likert scale, ranging from ‘strongly disagree’ to ‘strongly agree’. The Italian version of the ERQ has a Cronbach's α coefficient of 0.84 for the reappraisal scale and 0.72 for the suppression scale.

### 2.3 Procedure

Data were collected through an online survey created with Google Forms. After providing informed consent, participants completed demographic and anamnestic questions, followed by the standardized questionnaires. A description of SP was presented and participants were asked whether they had ever experienced a similar episode (‘Some people have had the experience …’). Those who responded affirmatively completed the Unusual Sleep Experiences Questionnaire (USEQ); those who responded negatively were directed to skip directly to the next questionnaires. The survey required approximately 25–30 min to complete. Participants were automatically classified into two groups: individuals who reported at least one lifetime episode of SP (SP+) and those who had never experienced SP (SP−). The study was conducted in accordance with the Declaration of Helsinki and approved by the Comitato Etico per la Ricerca Transdisciplinare (CERT) of the University of Rome Sapienza (protocol ID: CERT_1973F10DB35). All participants provided informed consent before participation.

### 2.4 Statistical Analyses

All analyses were conducted using Jamovi (version 2.3.28). Data were screened for normality using the Shapiro–Wilk test and for homogeneity of variances using Levene's test. Between‐group differences (SP+ vs. SP–) were initially explored using analyses of variance (ANOVA). When demographic variables (e.g., age) differed significantly between groups or were significantly associated with the dependent variables, analyses of covariance (ANCOVA) were conducted to adjust for potential confounding effects. Effect sizes were reported as partial eta squared (*ηp* ^2^). To account for multiple comparisons, *p* values were adjusted using the False Discovery Rate (FDR; Benjamini–Hochberg procedure). Statistical significance was set at *p* < 0.05 (two‐tailed). Descriptive statistics are reported as means ± standard deviations.

## 3 Results

### 3.1 Descriptive Statistics

The final sample comprised 210 participants (64.3% female; M = 43.9, SD = 14.5). Of these, 55 individuals (26.2%) reported experiencing at least one lifetime episode of sleep paralysis (SP+), whereas 155 (73.8%) had never experienced SP (SP–). No significant association emerged between gender and SP condition, *χ* ^2^(2) = 0.71, *p* = 0.70. However, participants in the SP+ group were significantly younger than those in the SP– group (*t*(208) = 3.05, *p* = 0.003, *d* = 0.43). Descriptive characteristics for the total sample and by SP condition are reported in Table 1.

**TABLE 1:** Sample characteristics.

	Total sample (*N* = 210)	SP –(*N* = 155)	SP + (*N* = 55)
Gender (*N*; %)			
Male	73 (34.8%)	53 (34.2%)	20 (36.4%)
Female	135 (64.3%)	101 (65.2%)	34 (61.8%)
Other	2 (1.0%)	1 (0.6%)	1 (2%)
Age M (SD)	43.9 (14.5)	45.7 (14.9)	38.8 (12.2)
Education (*N*; %)			
Middle school	8 (3.8%)	6 (4.0%)	2 (3.6%)
High school diploma	56 (26.7%)	37 (24.0%)	19 (34.5%)
Bachelor's degree	100 (47.6%)	81 (52.2%)	19 (34.5%)
Postgraduate	46 (21.9%)	31 (20.0%)	15 (27.2%)
Marital status (*N*; %)			
In a relationship	168 (80.0%)	124 (80.0%)	44 (80.0%)
Single	42 (20.0%)	31 (20.0%)	11 (20.0%)
Employment status (*N*; %)			
Employed	151 (71.9%)	108 (80%)	43 (78.2%)
Student	23 (11.0%)	18 (11.6%)	5 (9.1%)
Retired	24 (11.4%)	20 (13.0%)	4 (7.3%)
Unemployed	12 (5.7%)	9 (6.0%)	3 (5.5%)
How often do you sleep with someone? (*N*; %)			
Never	42 (20%)	30 (19.4%)	12 (22%)
Always	120 (57.1%)	92 (59.4%)	28 (51.0%)
4–6 times/week	16 (7.6%)	10 (6.5%)	6 (11.0%)
3 times/week	7 (3.3%)	5 (3.2%)	2 (4.0%)
1–2 times/week	25 (11.9%)	18 (11.6%)	7 (13.0%)
Preferred sleeping position (*N*; %)			
Right side	75 (35.7%)	56 (36.1%)	19 (34.5%)
Left side	80 (38.1%)	60 (38.7%)	20 (36.4%)
Prone	38 (18.1%)	27 (17.4%)	11 (20.0%)
Supine	17 (8.1%)	12 (7.7%)	5 (9.1%)
Have you ever suffered from psychiatric disorders? (*N*; %)			
No	140 (66.7%)	105 (67.7%)	35 (64.0%)
Yes	70 (33.3%)	50 (32.3%)	20 (36.4%)
Have you ever suffered from neurological disorders? (*N*; %)			
No	206 (98.1%)	152 (98.0%)	54 (98.2%)
Yes	4 (1.9%)	3 (2.0%)	1 (2.0%)
Have you ever suffered from sleep disorders? (*N*; %)			
No	115 (54.8%)	102 (66.0%)	13 (23.6%)
Yes	95 (45.2%)	53 (34.2%)	42 (76.4%)
Have you ever suffered from chronic pain? (*N*; %)			
No	167 (79.5%)	123 (79.4%)	44 (80.0%)
Yes	43 (20.5%)	32 (21.0%)	11 (20.0%)

Abbreviations: M = mean; *N* = number; SD = standard deviation; SP− = no sleep paralysis; SP+ = sleep paralysis.

### 3.2 USEQ Statistics

Among the 55 participants reporting SP, 43.6% had not experienced an episode in the past year, and 80% had not experienced one in the past month. The mean age of onset for the first episode was 22.0 years (SD = 10.14), with a mean interval of 10.2 years (SD = 8.78) before the study. Regarding the timing of the most recent episode, 7.3% reported one within the past week, 12.7% within the past month, 38.2% within the past year and 41.8% more than 1 year ago.

### 3.3 Group Comparison

Given the significant age difference between groups (see Table 2), ANCOVA analyses controlling for age were conducted to compare SP+ and SP– groups on all psychological and sleep‐related variables. Participants with SP reported significantly greater insomnia severity compared to those without SP (*F*(1,152) = 8.528, *p* = 0.004). Significant differences emerged in sleep disturbance (*F*(1,152) = 4.272, *p* = 0.04); no difference emerged in global PSQI score or in other components, such as sleep duration, latency, or efficiency. Similarly, emotion regulation strategies (ERQ), perceived stress (PSS) and trait anxiety (STAI‐T) did not differ between groups. Regarding interoceptive sensitivity (MAIA), the SP+ group reported significantly higher scores on the following subscales: noticing (*F*(1,152) = 3.665, *p* = 0.04), attentional regulation (*F*(1,152) = 9.573, *p* = 0.002), self‐regulation (*F*(1,152) = 8.654, *p* = 0.004), body listening (*F*(1,152) = 5.503, *p* = 0.02) and trusting (*F*(1,152) = 4.249; *p* = 0.04; see Figure 1). Interestingly, higher cognitive pre‐sleep arousal and elevated PSQI‐A scores were initially observed, but these effects were no longer significant after controlling for the covariate. No other significant differences were found between groups (see Table 2).

**TABLE 2:** Results of the test performed on the various variables considered.

Variable	SP−		SP+		*F*	*p*	*Partial eta*
	M	SD	M	SD			
Age	45.66	14.87	38.84	12.2	9.33	0.003	0.04
ERQ							
Cognitive reappraisal	4.38	1.15	4.27	1.13	< 1	0.51	0.001
Expressive suppression	3.44	1.21	3.56	1.35	< 1	0.50	0.002
PSS	16.66	6.73	18.02	7.62	< 1	0.98	0.001
PSAS							
Cognitive vigilance	17.10	5.69	18.86	5.75	1.48	0.22	0.007
Somatic vigilance	11.08	3.47	12.16	4.77	1.47	0.21	0.007
MAIA							
Noticing	2.53	1.39	3.04	1.38	3.66	0.04	0.02
Not‐distracting	2.39	1.03	2.25	0.95	1.58	0.21	0.02
Not‐worrying	2.75	1.16	2.75	1.27	< 1	0.65	0.001
Attentional regulation	2.55	1.21	3.11	1.06	9.57	0.002	0.05
Emotional awareness	2.89	1.26	3.26	1.31	3.07	0.08	0.01
Self‐regulation	2.41	1.22	2.87	1.19	8.65	0.004	0.04
Body listening	2.20	1.26	2.66	1.31	5.50	0.02	0.03
Trusting	2.86	1.42	3.27	1.40	4.24	0.04	0.02
ISI	12.81	4.48	14.65	3.97	8.52	0.004	0.04
STAI‐T	49.99	3.01	49.60	3.11	< 1	0.94	0.001
PSQI							
Subjective sleep quality	1.15	0.59	1.20	0.68	< 1	0.59	0.001
Sleep latency	1.08	0.97	1.04	0.88	< 1	0.63	0.002
Sleep duration	0.94	0.67	0.96	0.79	< 1	0.36	0.001
Habitual sleep efficiency	0.54	0.85	0.60	0.95	< 1	0.45	0.001
Sleep disturbances	1.32	0.55	1.49	0.72	4.27	0.04	0.02
Sleep medications	0.40	0.94	0.22	0.74	< 1	0.41	0.001
Daytime disfunction	0.92	0.72	1.02	0.71	< 1	0.59	0.001
PSQI scores	6.35	3.20	6.53	3.02	< 1	0.41	0.002
PSQI‐A	4.30	4.06	5.70	4.90	1.46	0.22	0.009

*Note:* Results are reported considering FDR‐adjusted values adjusted for age.

Abbreviations: df = degree of freedom; ERQ = Emotional Regulation Questionnaire; ISI = Insomnia Severity Index; M = mean; MAIA = Multidimensional Assessment of Interoceptive Awareness; PSAS = Pre‐Sleep Arousal Scale; PSQI = Pittsburgh Sleep Quality Index; PSQI‐A = Pittsburgh Sleep Quality Index—Addendum; PSS = Perceived Stress Scale; SD = standard deviation; SP− = no sleep paralysis; SP+ = sleep paralysis; STAI‐T = State–Trait Anxiety Inventory—Trait.

**FIGURE 1:**
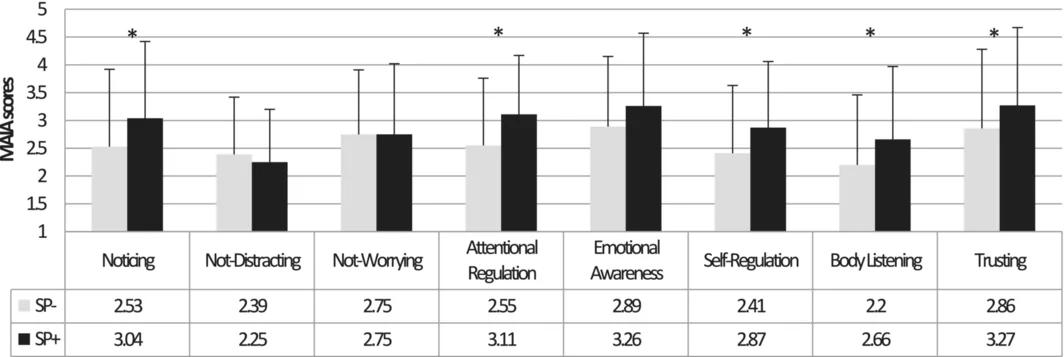
Between‐group differences (SP+ and SP−) in MAIA scores. Error bars represent the standard error of the mean.

## 4 Discussion

The first aim of this study was to investigate SP, focusing on its prevalence and characteristics. Our study revealed a lifetime prevalence of SP of 26.2%, a figure significantly higher than the 7.6% reported in previous systematic reviews (Sharpless and Barber 2011). This discrepancy may be attributed to selection bias, as participants were recruited via advertisements explicitly mentioning SP, which may have attracted individuals with prior experience of this phenomenon. Additionally, 33.3% of our sample had psychiatric disorders, which are known to correlate with increased SP frequency (Sharpless and Barber 2011). A more detailed examination of the specific psychiatric conditions present in our sample would be beneficial for understanding their interaction with SP experiences. The mean age of onset for the first SP episode was 21 years, aligning with physiological sleep variations typical of late adolescence (Colrain and Baker 2011). Although the age of onset observed in this study is consistent with developmental changes in sleep architecture, it is likely that behavioural, environmental and lifestyle factors also contribute to the emergence of SP during this period. In interpreting the phenomenological characteristics of SP, it is important to consider the potential influence of sociocultural factors. In the present study, the relatively low prevalence of folkloric interpretations may be partly explained by the high educational level of the sample, which could have favoured more scientifically grounded or culturally normative explanations of the experience. This may have reduced the tendency to interpret SP episodes through traditional or supernatural frameworks, thereby influencing how participants reported and conceptualized their experiences.

A novel aspect emerged from the investigation of episode repetition upon re‐sleeping in the same position: 32.7% of participants reported this occurrence, suggesting a potential role for proprioception in SP experiences that should be further explored using standardized techniques.

### 4.1 Symptomatology and Hallucinatory Features

The symptom profile of SP was consistent with previous findings (Colombo and Cellini 2024), with the inability to speak (83.6%), numbness (76.3%) and the inability to open eyes (60%) being the most common manifestations. These findings are consistent with the muscle atonia characteristic of REM sleep. The hallucinatory component of SP episodes showed variability, consistent with prior research (Jalal et al. 2020; Jalal and Hinton 2013; Ohaeri et al. 2004; Hinton et al. 2005). 65.4% of participants reported intrusive‐type hallucinations, supporting Cheyne's tripartite model Cheyne (2002), which categorizes SP hallucinations into intruder (65.4%), incubus (52.7%) and vestibular‐motor (45.4%) experiences. The sensation of chest pressure, often described as ‘a weight’ (61.3%) or ‘a person sitting on the chest’ (35.5%), was also consistent with Colombo & Cellini's results Colombo and Cellini (2024). Visual hallucinations were predominantly humanoid figures (87.7%), while auditory hallucinations frequently included human voices (37%) and footsteps (21%), in line with previous studies (Colombo and Cellini 2024; Cheyne et al. 1999; Sharpless and Kliková 2019). Notably, 83.6% of participants reported intense fear during SP episodes, a higher percentage than in some studies (Cheyne et al. 1999; Colombo and Cellini 2024), though similar to findings from specific samples (Sharpless et al. 2010). Analysis of fear intensity over time revealed a gradual decrease of SP across successive episodes, suggesting a potential adaptation process. Interestingly, folkloric interpretations of SP were rare, with low reports of figures such as Janara or Pantafica and minimal references to alien abductions. This may reflect increased awareness of the scientific nature of SP, as well as cultural differences in interpretation. Further research should examine Italian cultural beliefs about SP to deepen understanding of its sociocultural context.

### 4.2 Insomnia and Sleep Disturbances: A Hyperactivation Explanation

Consistent with the literature, our study found that participants who had experienced at least one episode of SP reported higher levels of pre‐sleep arousal, particularly in the cognitive and somatic arousal dimensions of the PSAS, compared to those who had never experienced SP. This finding is consistent with empirical results showing higher arousal levels before SP episodes than during normal sleep (Takeuchi et al. 2002), as well as with hypotheses suggesting a key role for pre‐sleep hyperarousal in individuals with SP (Denis et al. 2018). However, when controlling for a relevant covariate (e.g., age), these differences were attenuated and no longer reached statistical significance, suggesting that the observed effects may be partially explained by demographic or other confounding factors.

Additionally, we observed that individuals who experienced SP reported more severe insomnia symptoms than those who had never encountered it, a finding consistent with previous studies (Denis et al. 2018; Otsuka et al. 2017; Ohayon et al. 1999). Importantly, significant differences also emerged in sleep disturbance, suggesting greater instability in sleep continuity among individuals with SP. Notably, the increase in pre‐sleep arousal in SP subjects, although initially observed, was attenuated after controlling for covariates and should therefore be interpreted with caution. Nonetheless, this pattern remains conceptually consistent with that observed in insomnia patients, who exhibit higher beta activity before sleep onset (Freedman 1986; Perlis et al. 2001) and even during REM sleep (Merica et al. 1998; Freedman 1986). This suggests that cortical hyperarousal, or ‘cognitive arousal’ (Perlis et al. 2001; Bastien et al. 2009), may be a shared characteristic, potentially reflecting interrupted cortical networks attempting to initiate or resume sleep (Perlis et al. 2001; Bastien et al. 2009). SP also shares characteristics with other unusual dream experiences, such as nightmares, vivid dreams, lucid dreams and hypnagogic hallucinations (Lišková et al. 2016; Simor, Bódizs, et al. 2013; Simor, Horváth, et al. 2013; Drinkwater et al. 2020). For instance, nightmares are associated with increased alpha activity during REM, indicating the intrusion of wakefulness (Simor, Bódizs, et al. 2013; Simor, Horváth, et al. 2013). Experimental studies inducing SP through sleep interruption (Takeuchi et al. 1992, 2002) have shown that SP episodes exhibit a ‘mixed’ state of consciousness, blending wakefulness and REM elements. EEG recordings during SP reveal alpha activity alongside typical REM patterns. Simor, Bódizs, et al. (2013); Simor, Horváth, et al. (2013) suggest that alpha oscillations during REM may represent brief periods of sleep instability, facilitating brain‐environment connectivity. This could be attributed to pre‐sleep hyperarousal intruding into the anticipated REM period. Moreover, it has been demonstrated that sleep deprivation can induce SP episodes, facilitating Sleep‐Onset REM Periods (SOREMPs), which are direct transitions from wakefulness to REM sleep (Takeuchi et al. 1992, 2002). This phenomenon was initially observed in narcoleptic patients (Hishikawa and Kaneko 1965; Hishikawa 1978) and later confirmed in healthy individuals subjected to sleep deprivation (Takeuchi et al. 1992, 2002). Within this framework, sleep disturbance and insomnia symptoms may contribute to the occurrence of SOREMPs, thereby increasing the likelihood of SP episodes. These findings are consistent with Hobson's AIM state‐space model, which posits SP as a blend of wakefulness and REM, characterized by high arousal, mixed internal and external inputs and mixed neuromodulation. PSG evidence supports high neural activation (Denis et al. 2018) and the co‐occurrence of hallucinations (internal input) and environmental awareness (external input) during SP further validates this model (D'Agostino and Limosani 2008). However, these findings should be interpreted cautiously, as some effects were marginal and may not survive correction for multiple comparisons.

Contrary to the data presented in the literature, our results show that subjects who had experienced at least one episode of SP at some point in their lives did not have a worse sleep quality than those who had never experienced it. In fact, there were no statistically significant differences in the total PSQI scores, despite increased sleep disturbance in the SP group. Notably, increased sleep disturbances in the absence of differences in overall sleep quality may reflect a form of chronic nocturnal hyperactivation, in which individuals experience discrete sleep disruptions while maintaining a relatively stable subjective perception of sleep, possibly indicating a process of adaptation to persistent sleep‐related difficulties. We could attribute this anomaly to several factors. First, it is essential to remember that the PSQI is an instrument that measures subjective sleep quality over the last month, rather than objective sleep characteristics. In the group of subjects with SP, 80% (*n* = 44) reported not having had any episode of SP during the last month, and this could be a factor that contributed to ‘improving’ the quality of sleep of the subjects in the SP group, precisely in the period in which the PSQI was detected. In addition, in the control group, consisting of subjects who had never experienced episodes of SP during their lives, 34.2% of respondents reported suffering or having suffered from sleep disorders. The presence of self‐reported sleep disorders in a third of subjects without paralysis could have contributed to ‘blurring’ the difference between the two groups in the global PSQI score. Perhaps the most significant factor contributing to the obscuring of this difference between the two groups is that our total sample is predominantly composed of women (64.3%), with an average age of 44 years (SD = 14.5). It is known that during this period, a perimenopausal phase may begin (40–45 years), during which one of the main symptoms of this condition is a worsening of sleep quality (Baker et al. 2018). The relevant presence of this condition among the respondents may have affected the results.

### 4.3 Sleep Paralysis and Interoception: The Role of the Body

A pioneering aspect of our study is the preliminary evidence of a significant association between interoceptive sensitivity and SP. While prior research has established connections between heightened interoceptive awareness and conditions such as insomnia and nightmare disorders (Arora et al. 2021; Wei et al. 2016; Perogamvros et al. 2019; Faccini et al. 2023), our work is the first to illuminate its role in SP. Interoception, the intricate process by which the nervous system perceives, interprets and integrates signals from the body, provides a moment‐to‐moment map of our internal physiological landscape (Khalsa et al. 2018). In our investigation, individuals with a history of SP exhibited markedly elevated self‐reported interoceptive sensitivity, particularly within key dimensions of the MAIA. These dimensions included heightened noticing of bodily sensations, enhanced attention regulation, increased self‐regulation, greater emotional awareness and amplified body listening. Notably, these individuals demonstrated a profound attunement to their corporeal experiences, reflecting a heightened awareness of the body's internal signals. The increased perception and awareness of both pleasant and unpleasant bodily sensations, coupled with enhanced emotional awareness, suggest a potential state of bodily hypersensitivity in individuals prone to SP. This heightened focus on the body's internal cues, while seemingly indicative of adaptive self‐monitoring, may paradoxically contribute to a state of hyperarousal conducive to SP episodes. The ability to regulate emotional distress through attention to bodily sensations is typically a beneficial skill, but it may exacerbate the development of SP episodes in people who usually experience them. Although the cross‐sectional design of our study precludes definitive conclusions regarding causality, a heightened focus on interoceptive stimuli is associated with higher levels of pre‐sleep arousal, thereby disrupting sleep initiation and maintenance. This disruption could, in turn, increase susceptibility to SP episodes. This conceptualization aligns with observations in insomnia, where attentional biases towards internal and external stimuli contribute to heightened arousal (Harvey and Payne 2002; Espie et al. 2006). The implications of this novel finding are significant. By highlighting the role of interoception in SP, we open new avenues for research and potential therapeutic interventions. Future studies could investigate the effectiveness of interoceptive training or mindfulness‐based practices in reducing the frequency and severity of SP episodes. Furthermore, this discovery highlights the importance of considering the body's role in the pathogenesis of SP, a phenomenon that has traditionally been viewed primarily through a psychological lens. Importantly, interoception in this study was assessed through self‐report (MAIA), reflecting interoceptive sensibility rather than objective interoceptive accuracy. Therefore, conclusions regarding bodily sensitivity should be interpreted within this framework.

## 5 Limitations

Although our study has yielded very interesting results, given its cross‐sectional design, no causal inferences can be drawn regarding the observed relationships, and the directionality of these associations remains unclear. Some limitations need to be considered. First, the study was largely exploratory in nature and considered many available variables. Therefore, independent replication of these results is essential. Second, given the cross‐sectional design, it is impossible to assess the direction of the observed relationships. For example, although initial analyses suggested that subjects who had experienced at least one episode of SP reported higher arousal before sleep onset, this effect was attenuated after controlling for covariates and should therefore be interpreted with caution. It remains unclear whether pre‐sleep arousal represents a causal factor or reflects the influence of additional variables. Future longitudinal designs are needed to clarify the nature of these relationships. Furthermore, in our study, only subjective sleep variables were assessed. While this provided for a good sample size, it is well known that subjective and objective measures of sleep do not always align (Zhang and Zhao 2007). Polysomnography (PSG) is considered the gold standard in sleep assessment; however, its greater accuracy comes at the expense of smaller sample sizes. Although laboratory investigations of SP have been conducted, they are limited by the relative rarity of SP episodes, making it difficult to study this phenomenon in a laboratory setting (Takeuchi et al. 1992; Walther and Schulz 2004). Future research should also incorporate a more comprehensive assessment of psychiatric symptoms, as a broader range of psychological conditions may be associated with sleep quality and could further contribute to understanding SP (Casagrande, Favieri, Langher, et al. 2020; Casagrande, Favieri, Guarino, et al. 2020; Corbo et al. 2023).

Finally, a dichotomous variable was used to measure SP for statistical reasons. However, this approach reduces sensitivity. It is debatable whether a person who experiences SP only a few times in their life has the same risk factors for experiencing episodes as someone who experiences SP nearly every night. Moreover, reliance on self‐report and retrospective recall may bias estimates of frequency and phenomenology. The accuracy of recall for episodes that occurred long ago should also be taken into account, and prospective measures of SP should be more readily employed in future studies.

## 6 Conclusions

While some aspects of our findings diverge from previous research on SP, this study provides initial evidence for a link between interoceptive sensitivity and SP. Our results indicate that individuals with a history of these episodes show greater interoceptive sensitivity, reflecting a heightened awareness of internal bodily signals. This finding introduces a novel perspective to the study of SP, which has traditionally been approached mainly from psychological viewpoints. Our results suggest that physiological processes, particularly interoceptive sensitivity, also play a meaningful role. The elevated interoceptive sensitivity observed in individuals with SP may influence their capacity to regulate pre‐sleep arousal, potentially increasing vulnerability to these episodes. Future research should investigate whether interventions designed to modulate interoceptive sensitivity, such as mindfulness‐based or biofeedback approaches, can support better regulation of pre‐sleep arousal and reduce SP frequency. Overall, our study highlights the relevance of considering interoception as a crucial component in understanding and managing SP, thereby opening up new avenues for research and clinical exploration.

## Author Contributions

**Giuseppe Forte:** conceptualization, methodology, formal analysis, writing – review and editing, writing – original draft, data curation. **Danilo Giaccari:** conceptualization, investigation, validation, data curation, writing – original draft, writing – review and editing. **Francesca Favieri:** conceptualization, writing – original draft, writing – review and editing, formal analysis. **Maria Casagrande:** conceptualization, writing – original draft, writing – review and editing, supervision.

## Funding

The authors have nothing to report.

## Conflicts of Interest

The authors declare no conflicts of interest.

## Data Availability

The data that support the findings of this study are available on request from the corresponding author. The data are not publicly available due to privacy or ethical restrictions.
